# Causes of Death among Commercially Insured Multiple Sclerosis Patients in the United States

**DOI:** 10.1371/journal.pone.0105207

**Published:** 2014-08-21

**Authors:** Douglas S. Goodin, Michael Corwin, David Kaufman, Howard Golub, Shoshana Reshef, Mark J. Rametta, Volker Knappertz, Gary Cutter, Dirk Pleimes

**Affiliations:** 1 Department of Neurology, University of California San Francisco, San Francisco, California, United States of America; 2 Slone Epidemiology Center at Boston University, Boston, Massachusetts, United States of America; 3 Care-Safe, Boston, Massachusetts, United States of America; 4 Bayer HealthCare Pharmaceuticals, Whippany, New Jersey, United States of America; 5 Teva Pharmaceuticals, Frazer, Pennsylvania, United States of America, Department of Neurology, Heinrich Heine University, Dusseldorf, Germany; 6 University of Alabama School of Public Health, Birmingham, Alabama, United States of America; 7 Myelo Therapeutics GmbH, Berlin, Germany; National Institutes of Health, United States of America

## Abstract

**Background:**

Information on causes of death (CODs) for patients with multiple sclerosis (MS) in the United States is sparse and limited by standard categorizations of underlying and immediate CODs on death certificates. Prior research indicated that excess mortality among MS patients was largely due to greater mortality from infectious, cardiovascular, or pulmonary causes.

**Objective:**

To analyze disease categories in order to gain insight to pathways, which lead directly to death in MS patients.

**Methods:**

Commercially insured MS patients enrolled in the OptumInsight Research database between 1996 and 2009 were matched to non-MS comparators on age/residence at index year and sex. The cause most-directly leading to death from the death certificate, referred to as the “principal” COD, was determined using an algorithm to minimize the selection of either MS or cardiac/pulmonary arrest as the COD. Principal CODs were categorized into MS, cancer, cardiovascular, infectious, suicide, accidental, pulmonary, other, or unknown. Infectious, cardiovascular, and pulmonary CODs were further subcategorized.

**Results:**

30,402 MS patients were matched to 89,818 controls, with mortality rates of 899 and 446 deaths/100,000 person-years, respectively. Excluding MS, differences in mortality rate between MS patients and non-MS comparators were largely attributable to infections, cardiovascular causes, and pulmonary problems. Of the 95 excessive deaths (per 100,000 person-years) related to infectious causes, 41 (43.2%) were due to pulmonary infections and 45 (47.4%) were attributed to sepsis. Of the 46 excessive deaths (per 100,000 person-years) related to pulmonary causes, 27 (58.7%) were due to aspiration. No single diagnostic entity predominated for the 60 excessive deaths (per 100,000 person-years) attributable to cardiac CODs.

**Conclusions:**

The principal COD algorithm improved on other methods of determining COD in MS patients from death certificates. A greater awareness of the common CODs in MS patients will allow physicians to anticipate potential problems and, thereby, improve the care that they provide.

## Introduction

Multiple sclerosis (MS) is a chronic disease of the central nervous system in which there are recurrent injuries both to the myelin sheaths that surround the nerve axons and also, to a lesser extent, to the axons themselves. [1] Although it is often said that MS does not affect mortality, the available data suggest that patients with MS have a life expectancy that is decreased by about 5–10 years compared to the general population when matched for age and sex. [2]–[7].

**Figure 1:**
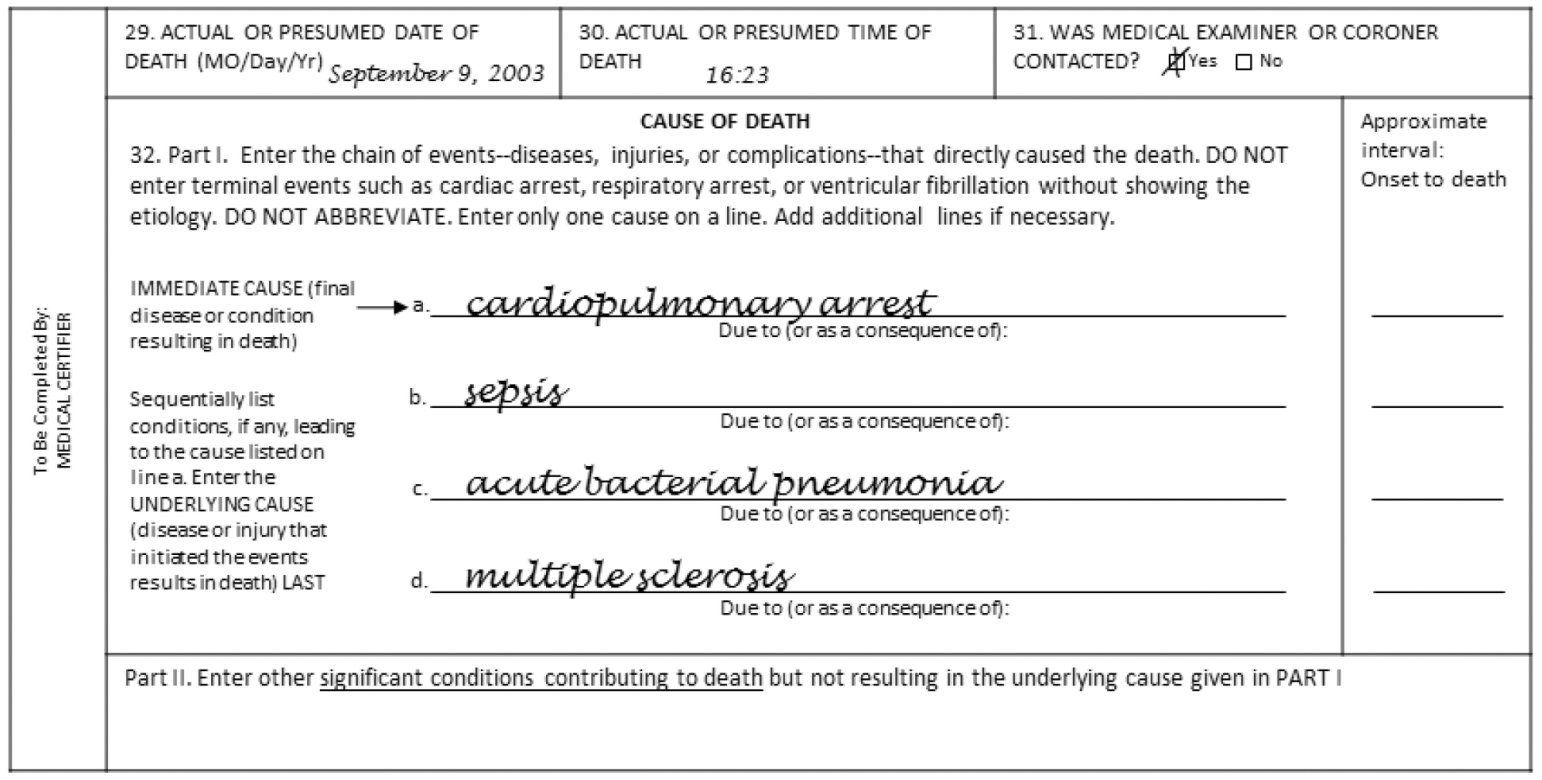
Sample death certificate. A hypothetical death certificate for a deceased patients with multiple sclerosis (MS) based on the standard death certificate format. [8] For this patient, the immediate cause of death (COD) would be cardiopulmonary arrest and the underlying COD would be MS. However, sepsis would be considered the principal COD.

Only limited data are available regarding the causes of death (CODs) that account for the excessive mortality found in the MS population. In part, this is due to inherent limitations that arise from the manner in which CODs are documented on death certificates. Death certificates typically have 5 positions for categorizing a death (Figure 1). [8], [9] In Part I, the first position (the immediate COD) is meant to be the condition (eg, hepatic encephalopathy) that led directly to the death. [9] The next 3 positions (the underlying CODs) are for those causes that led sequentially to the immediate cause (eg, chronic alcoholism leading to cirrhosis of the liver leading to the hepatic encephalopathy). [9] The fifth position (Part II), is for significant conditions, contributing to the death but not leading directly to the underlying causes (eg, malnutrition, ascites, etc). [9].

The use of death certificate data leads to some inaccuracies in establishing the relationship between MS and specific CODs. For example, the use of the underlying COD (as is typical for the reporting of vital statistics) is often inaccurate because MS itself is frequently listed as the sole underlying cause, and, as such, this entry provides no insight into whether a particular complication of end-stage MS (eg, an infection) may have actually led to the death rather than MS itself. Similarly, the use of the immediate COD may result in other errors. For example, many physicians list cardiac or respiratory arrest as the immediate COD in the first position on the death certificate (Figure 1). [10] Nevertheless, because these entities represent the final common pathway of all deaths, they are not helpful for understanding what condition actually led directly to the death (eg, pneumonia, myocardial infarction, gunshot wound, etc).

We recently reported the results from a retrospective cohort study in which we compared survival and mortality patterns in patients with MS drawn from the OptumInsight Research (OIR) database. [11] This database contains the billing/claims data from a national commercial health insurance plan in the United States (US). [11] We used a matched cohort design, including both patients with MS and non-MS comparators from the same source population and analyzed mortality data covering the period of 1996–2009. [11] In this study, we were able to show that mortality rate among patients with MS was approximately twice that observed among matched controls. [11].

In the present study, we attempted to gain insight into those CODs, which contribute to the excessive mortality seen in MS patients. To accomplish this, we developed a novel algorithm for identifying (from death certificate data) the cause most directly leading to the death. We refer to this as the “principal” COD. We compared our new principal COD algorithm with standard death certificate–based definitions using the underlying and immediate COD assignments to evaluate the extent to which this new method might provide improved COD information among individuals with a chronic illness such as MS.

## Methods

The project utilized a commercially available database, anonymous with respect to any individual-identifying information, as well as publically available death certificate data. Individual institutional approval was, therefore, unnecessary.

### Selection of Subjects and Determination of Mortality – OIR Claims Database

Subjects were drawn from the OIR health-care claims database. The database contains billing claims information for over 39 million individuals insured through United HealthCare; there are approximately 15 million covered lives per year and 7.5 million patients with lab results. [12] The database is geographically diverse and is representative of the commercially insured population of the US. [12].

Patients with MS were selected for inclusion in the study if they met the following criteria:

- Inclusion in the database for ≥3 months during the period from 1996–2009 – these years were selected to cover the time from the start of the modern period of treatment with MS disease modifying therapy (DMT) to the most recently available data at the time of analysis.
- Age ≥18 years at the time of their first ICD9 diagnostic code of 340 (the code for MS).
- At least 2 ICD9-340 diagnosis codes ≥30 days apart *or* at least 1 ICD9 diagnostic code of 340 and ≥1 billing code for DMT, defined as any of the following drugs: interferon beta-1a, interferon beta-1b, glatiramer acetate, or natalizumab. The reason for limiting consideration to only these agents was because these are MS-specific therapies that were available during the time period covered by the analysis and, therefore, their use is likely to reflect a true MS diagnosis.

Similarly, control subjects were selected for inclusion if they met the following criteria:

- Inclusion in the database for ≥3 months and during the period from 1996–2009.
- The same age, sex, and residence region (using the US Census categories of Northeast, Midwest, South, West) during the index year (ie, the year of entry into the insurance database) as the matched patient with MS.

Up to 3 matched non-MS control subjects were selected for each patient with MS as described above.

Deaths among the selected subjects were identified by linkage with the National Death Index (NDI) and the Social Security Administration Death Master File (SSA DMF). Standard algorithms, which included Social Security number, were used to determine matches. A death was considered valid if it was identified through either source. Mortality information for each subject was searched through the end of 2009.

### Determination of Cause of Death

Determinations of COD were based solely from the information provided on the death certificate for those subjects whose death was identified by linkage with the NDI. This information was available for 1,451 (91.9%) of the deaths among patients with MS, and 2,127 (91.2%) of the deaths among non-MS controls. The information was not available for 128 (8.1%) deaths among patients with MS and 205 (8.8%) deaths among non-MS controls who were identified through the SSA DMF without NDI linkage. All deaths without death certificate information were categorized as being due to an unknown cause.

As shown in Figure 1, one of the ICD10 codes listed in Part 1 of the death certificate was assigned as the COD for each of the 3 approaches to COD assignment evaluated in this study; the immediate, the underlying, and the principal COD methods. The ICD10 codes for immediate and underlying COD were based on standard death certificate categorizations for Part 1 of the death certificate (Figure 1). The principal COD method used the ICD10 code at the top of Part 1 of the death certificate, with the following exceptions: ICD10 codes indicative of suicide were always considered the principal COD, regardless of its position on the death certificate; MS was considered the principal COD if the only ICD10 codes, which preceded the MS code on Part 1 of the death certificate (Figure 1), were those indicative of cardiac or respiratory arrest or both or if MS was the only code mentioned. Otherwise, MS was not considered the principal COD. Finally, ICD10 codes indicative of cardiac or respiratory arrest were only considered the principal COD if no other ICD10 codes (including MS) appeared on Part 1 of the death certificate.

**Figure 2:**
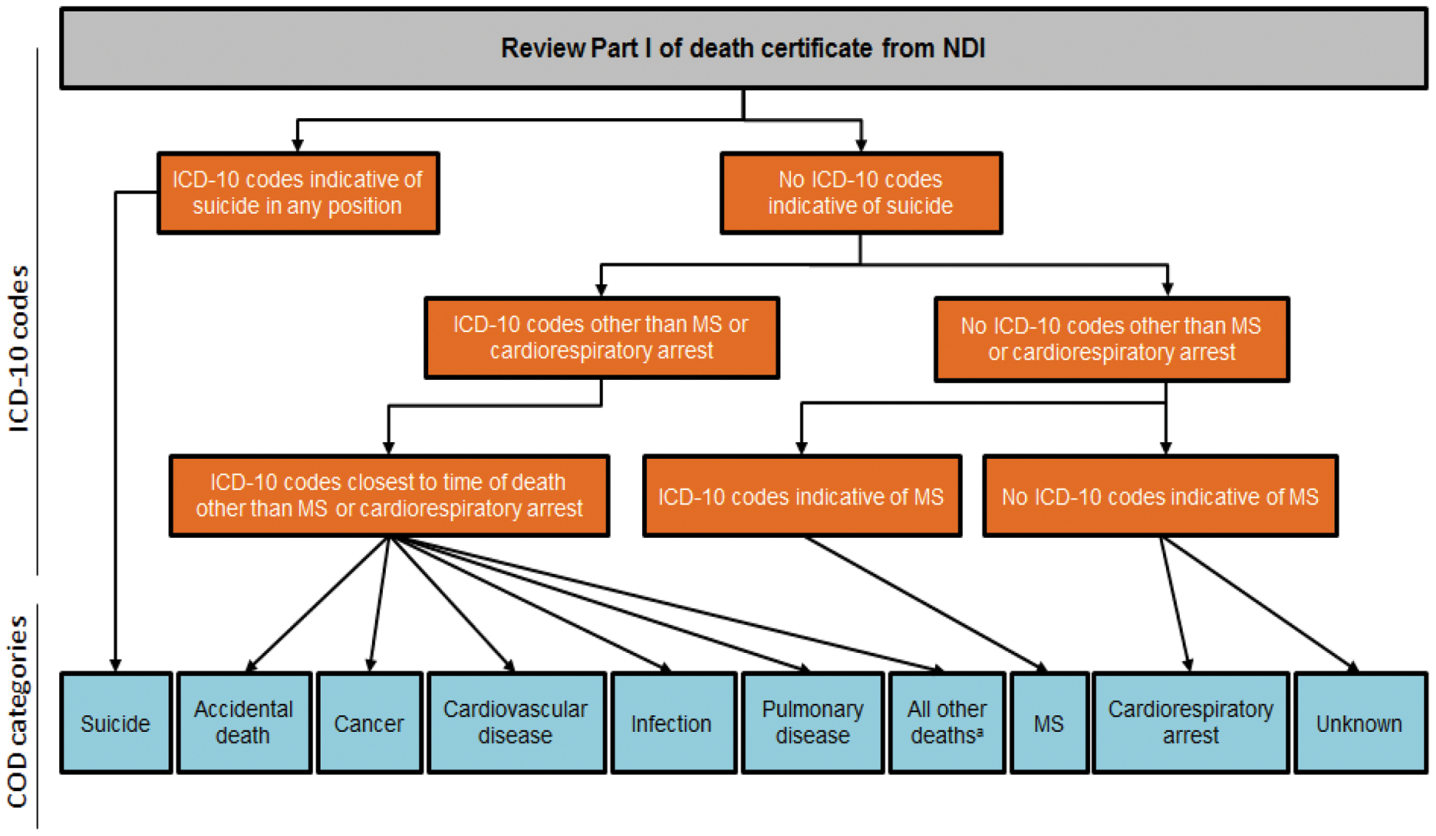
Algorithm for determining principal cause of death (COD) and primary disease/injury categories. The algorithm used to determine the principal COD based on ICD-10 codes. These codes were then sorted into 10 major disease/injury categories. ^a^Includes all ICD-10 codes not assigned to one of the other categories; examples include diabetes mellitus, all gastrointestinal diseases, hematologic diseases, etc.

Independently of the process for assigning ICD10 codes for each of the 3 COD methods, a 4-member author panel (consisting of a physician with experience in death certificate completion, an MS neurologist, and 2 general physicians) identified 7 principal disease/injury categories of COD. These are deaths due to: 1) cardiovascular disease, 2) cancer, 3) infections (including pulmonary infections), 4) pulmonary disease (excluding pulmonary infections), 5) MS, 6) accident, and 7) suicide. The panel then reviewed all the ICD10 codes noted in any position on death certificates and assigned them into 1 of these 7 categories. If a subject had ICD10 codes that did not fit into any of the prespecified categories (eg, codes for deaths due to diabetes, liver disease, kidney disease, etc), they were categorized as death due to “other known causes.” Death certificates indicating only cardiac or respiratory arrest were classified as death due to “cardiopulmonary arrest,” and deaths without any information were classified as death due to an “unknown cause” (Figure 2).

### Analytical Methods

Using the principal, underlying, and immediate COD methods, the overall and cause-specific mortality rate per 100,000 person-years with 95% confidence intervals (CI) were calculated for each category of death, and selected subcategories, in the MS and non-MS control populations. Differences in mortality rate per 100,000 person-years with 95% CI between patients with MS and non-MS controls were also calculated for each major category of death and selected subcategories.

## Results

### Accuracy of the MS Diagnosis

As discussed in the Methods, for the purposes of this study, the diagnosis of MS required either: a) ≥2 MS diagnostic codes or b) ≥1 MS diagnostic code plus a DMT prescription. The presence of a diagnostic code together with a DMT was considered to be sufficient evidence that the subject actually had MS. To determine the accuracy of the diagnosis when no DMTs were prescribed (36% of the total identified MS population), a medical chart review was conducted in 85 randomly selected such patients (thus demographically representative of the patients who did not receive DMTs). Sixty-two of these patients (73%) were judged to have definite MS –32 based on physician notes, 3 based on their use of DMTs despite not having a claim for this in the database, 26 based on both factors, and 1 based on the opinion of the neurologist reviewer. Therefore, assuming that the presence of an MS diagnostic code together with use of an MS-specific DMT accurately identified a patient as having MS, then the maximum expected false-positive rate was only 10% (0.37×0.27). Therefore, we concluded that the use of the “2-code” definition provided sufficient evidence that the subject actually had MS.

### Description of the Study Population

De-identified data for 30,436 patients with MS and 90,123 controls who met the initial screening criteria for selection were provided by OIR (Table 1). When matching was performed according to Kaufman et al, [11] these procedures led to the exclusion of 34 patients with MS and 305 controls, resulting in 30,402 cases and 89,818 non-MS controls being available for our analyses (Table 1). Of these, 29,411 cases (97%) had 3 matched controls; 594 (2%) had 2, and 397 (1%) had 1.

**Table 1:** Distribution of patients with MS and non-MS comparators.

		OIR Database		
		MS	Comparator	%^a^
		(n = 30,402)	(n = 89,818)	
Sex	Female	23,364	69,102	77
	Male	7,038	20,716	23
Mean (SD) years of age at index		44 (10.8)	44 (10.8)	–
Region	Northeast	3,546	10,395	12
	Midwest	9,822	28,816	32
	South	13,333	39,627	44
	West	3,701	10,980	12
Birth year	1920–1929^b^	335	964	1.1
	1930–1939	979	2,836	3.2
	1940–1949	4,578	13,381	15
	1950–1959	9,436	27,848	31
	1960–1969	8,998	26,727	30
	1970–1979	5,000	14,855	17
	1980–1989	1,060	3,159	3.5
	1990–1999	16	48	0.05
Mean insurance coverage interval	Total^c^	1,510	1,837	–
	Post-index^c^	1,071	610	–
	% time covered	92	86	–
DMT usage		19,359	–	64

a Due to the matching, the MS and comparator cohorts had the same distribution of demographic factors.

b The birth year for patients born before 1920 was set to 1920 to protect privacy.

c Includes gaps in coverage.

DMT, disease modifying therapy. MS, multiple sclerosis. OIR, OptumInsight Research.

Because of the matching procedures used, the distribution of demographic factors was identical for the MS and comparator cohorts. Each population was composed of 77% women and had a mean age at the index date of 44 years. The majority resided in either the Midwest (32%) or the South (44%), and almost all were born after 1920. The peak birth decades were the 1950s (31%) and 1960s (30%). The MS cohort had a somewhat shorter coverage period in the database than controls, but their post-index-date coverage was actually longer. The majority (64%) of patients with MS had ≥1 DMT prescription during their period of coverage.

A total of 1579 deaths (5.2%) were observed in the MS cohort, with 2332 deaths (2.6%) in non-MS comparators (Table 2). The overall mortality rate among the MS patients was approximately double that among non-MS controls (899 vs 446 per 100,000 person-years). The difference in mortality rate between the 2 cohorts was an excess of 453 deaths per 100,000 person-years in the MS population (Table 2). Further details of mortality rates according to sex and region have been published elsewhere. [11].

**Table 2:** Number of deaths and mortality rate/100,000 person-years (95% confidence interval) by major disease/injury category according to principal, underlying, and immediate cause of death (COD).

	Principal COD				Underlying COD				Immediate COD			
	MS		Non-MS comparators		MS		Non-MS comparators		MS		Non-MS comparators	
	n	Mortality rate	n	Mortality rate	n	Mortality rate	n	Mortality rate	n	Mortality rate	n	Mortality rate
Cardiovascular disease	325	185	652	125	284	162	595	114	259	147	531	101
		(165–206)		(115–135)		(143–182)		(105–123)		(130–167)		(93–110)
Cancer	183	104	557	106	221	126	686	131	166	95	486	93
		(90–120)		(98–116)		(110–144)		(121–141)		(81–110)		(85–102)
Infection	236	134	206	39	99	56	130	25	180	102	156	30
		(118–153)		(34–45)		(46–69)		(21–30)		(88–119)		(25–35)
Pulmonary disease	136	77	162	31	51	29	126	24	100	57	103	20
		(65–92)		(26–36)		(22–38)		(20–29)		(46–69)		(16–24)
MS	234	133	1^c^	0.2	502	286	1^c^	0.2	224	128	1^c^	0.2
		(117–151)		(0.0–1.1)		(261–312)		(0.0–1.1)		(111–145)		(0.0–1.1)
Accidental death	76	43	126	24	70	40	140	27	90	51	184	35
		(34–54)		(20–29)		(31–50)		(23–32)		(41–63)		(30–41)
Suicide	30	17	66	13	30	17	69	13	2	1.1	1	0.2
		(12–24)		(9.8–16)		(12–24)		(10–17)		(0.1–4.1)		(0.0–1.1)
All other deaths^a^	217	124	342	65	187	106	369	71	169	96	278	53
		(108–141)		(59–73)		(92–123)		(64–78)		(82–112)		(47–60)
Unknown^b^	129	73	208	40	128	73	207	40	129	73	208	40
		(61–87)		(34–46)		(61–87)		(34–45)		(61–87)		(34–46)
Cardiorespiratory arrest	13	7.4	12	2.3	7	4.0	9	1.7	260	148	384	73
		(3.9–13)		(1.2–4.0)		(1.6–8.2)		(0.8–3.3)		(131–167)		(66–81)
Overall	1579	899	2332	446	1579	899	2332	446	1579	899	2332	446)
		(855–945)		(428–464)		(855–945)		(428–464)		(855–945)		(428–464

a Includes all ICD-10 codes not assigned to one of the other categories; examples include diabetes mellitus, all gastrointestinal diseases, hematologic diseases, etc.

b Includes 333 deaths that did not have death certificate information because they were identified from Social Security records, but not from the national death index (NDI), plus a small number of individuals identified from the NDI whose death certificate data were insufficient to assign a cause of death.

c One subject in the comparator group developed MS post-entry into the study.

MS, multiple sclerosis.

#### Cause-specific mortality using principal, underlying, and immediate COD

As shown in Table 2, using the principal COD method, the categories with the highest mortality rate among patients with MS (other than MS itself) were cardiovascular disease (185 deaths per 100,000 person-years), infection (134 deaths per 100,000 person-years), cancer (104 deaths per 100,000 person-years), and pulmonary disease (77 deaths per 100,000 person-years). MS accounted for 133 deaths per 100,000 person-years, the third-highest mortality rate in the MS cohort. By contrast, among non-MS comparators, the categories with the highest mortality rate were: cardiovascular disease (125 deaths per 100,000 person-years), cancer (106 deaths per 100,000 person-years), infection (39 deaths per 100,000 person-years), and pulmonary disease (31 deaths per 100,000 person-years).

Among patients with MS, using the underlying COD from the death certificate, the disease/injury category with the highest mortality rate was MS, accounting for 286 of the 899 deaths per 100,000 person-years. Other CODs with relatively high mortality rates included cardiovascular disease and cancer. In contrast to the principal COD, the underlying COD indicated a higher mortality rate due to MS (133 vs 286 deaths per 100,000 person-years). The mortality rate due to infection (56 deaths per 100,000 person-years) was less than half of that attributed to infection by the principal COD (134 deaths per 100,000 person-years). The most common underlying CODs in the non-MS comparator cohort were cancer and cardiovascular disease.

The immediate COD indicated the highest mortality rate due to cardiorespiratory arrest, followed by cardiovascular disease, MS, and infection. Unlike the principal COD method, the immediate COD indicated the highest rate of mortality as a result of cardiorespiratory arrest (7.4 vs 148 deaths per 100,000 person-years). The mortality rate attributed to MS was similar using the principal and immediate COD approach (133 vs 128 deaths per 100,000 person years). Cardiovascular disease accounted for the highest mortality rate in the non-MS comparator population when using the immediate COD.

#### Differences in cause-specific mortality rate between the MS and comparator cohorts

**Figure 3:**
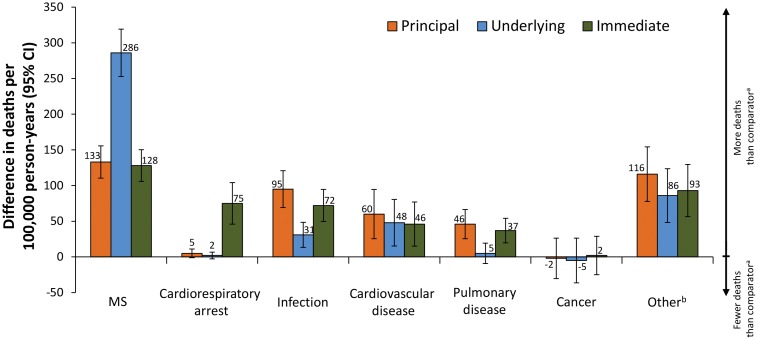
Difference in mortality rate (95% CI) between patients with MS relative to non-MS comparators by underlying, immediate, and principal COD. The difference in mortality rate between the MS and non-MS comparator cohorts using all 3 methods of determining COD. Positive values indicate disease/injury categories in which the MS cohort had a higher mortality rate. ^a^Comparator was a non-MS population matched for age, sex, and residence region. One subject in the comparator group developed MS post-entry into the study. ^b^Other includes suicide, accidental death, cancer, and those with an unknown COD.

The relative contribution of each major disease/injury category to the overall difference in mortality rate is illustrated in Figure 3. Based on the principal COD algorithm, the largest contributors to the difference in mortality rate between patients with MS and non-MS comparators (other than MS) included: infections (21.0% [95/453 deaths per 100,000 person-years]), cardiovascular disease (13.2% [60/453 deaths per 100,000 person-years]), and pulmonary disease (10.2% [46/453 deaths per 100,000 person-years]). Notably, all 3 of these COD categories had higher mortality rates when using the principal COD approach than either the underlying or the immediate COD approaches. Together, these 3 COD categories accounted for 44.4% of the total difference in mortality (201/453 deaths per 100,000 person-years). Moreover, using then the principal COD method, only 29.4% (133/453) of the difference was attributed to MS, and cardiac/respiratory arrest accounted for almost nothing. Nevertheless, despite the fact that these specific diseases accounted for the bulk of the excessive deaths observed, it is noteworthy that for every other disease category (with the exception of cancer), there was an excessive number of deaths in the MS population compared with the non-MS cohort.

Based on the underlying COD method, the differences in mortality rate between patients with MS and non-MS controls were mostly attributed to MS, which accounted for the difference in 63.1% (286/453) of the cases. Almost none of the difference was attributed to cardiac/respiratory arrest by this method. According to the immediate COD method, the differences in mortality rate between patients with MS and non-MS comparators were attributed to MS in 28.3% (128/453) of the cases and cardiac/respiratory arrest in 16.6% (75/453) of the cases.

#### Subcategory analysis of principal COD

**Figure 4:**
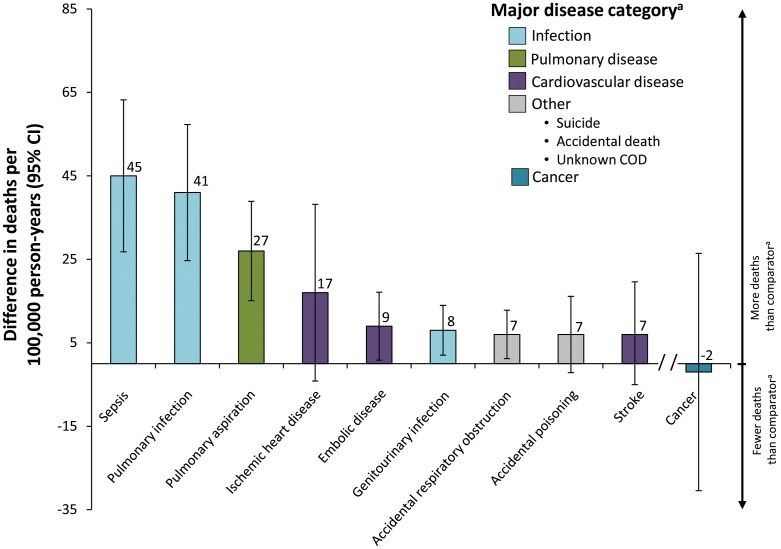
Contribution of subcategories of principal COD to differences in mortality rate (95% CI) between in patients with MS relative to non-MS comparators. Contribution of subcategories of principal COD to difference in mortality rate. Subcategories were only derived from the following main categories: infection, pulmonary disease, cardiovascular disease, and other. Cancer is shown only as a main category. ^a^Other principal COD subcategories evaluated but for which excess deaths in the MS cohort were <4/100,000 person-years included: accidental falls, asthma, chronic obstructive pulmonary disease, decubitus ulcers, dementia, diabetes, hepatic disease, paralytic disease, and renal disease.

Using the principal COD, subcategories of infection, pulmonary disease, and cardiovascular disease were assessed to gain greater insight into the categories that were particularly important contributors to the excessive mortality that MS populations experience (Figure 4). In the infection category, almost all of the excess mortality was attributed to either pulmonary infection or sepsis (90.6% [86/95 deaths per 100,000 person-years]). In the pulmonary disease category, more than half of the difference in excessive mortality was attributable to aspiration (58.7% [27/46 deaths per 100,000 person-years]). By contrast, within the category of cardiovascular disease, no single subcategory stood out as a contributor to the excess mortality.

## Discussion

In this study we demonstrated differences in the cause-specific mortality rate derived from the principal, the underlying, and the immediate COD reporting methods. An important goal of our study was to understand which non-MS conditions lead to the excess mortality in patients with MS. Using the principal COD method, it is apparent that infections, cardiovascular diseases, and pulmonary diseases are the most important contributors to the higher mortality rate seen among patients with MS (Figures 3 and 4). Moreover, analyses of subcategories of these principal COD categories indicated that certain disease states (eg, sepsis, pulmonary infection, and aspiration), which seem very likely to be intermediate steps on the pathway leading from advanced MS to death, were among the most important contributors to the excessive mortality observed among patients with MS (Figure 4). By contrast, and interestingly, death from suicide (which *a priori* we considered to be MS-related) was not significantly different between the MS and control populations (Table 2).

The main advantage of the principal COD method is that it decreases the number of cases in which cardiac/respiratory arrest or MS was the designated COD compared with the underlying and immediate COD methods. In fact, over 30% of the total mortality in the MS cohort was attributed to either MS or cardiac/respiratory arrest when using the underlying or the immediate COD approach (290 and 276 deaths per 100,000 person-years, respectively). The immediate and the principal COD methods estimated a similar mortality rate from MS itself (128 and 133 deaths per 100,000 person-years, respectively), a finding that suggests that if physicians place MS in the first position on the Part 1 of the death certificate, they almost never consider another condition to be underlying the death. Similarly, most patients (except those with rare brainstem lesions in vital locations) do not die directly from their MS. Therefore, when MS was listed as the immediate COD, it provided little information other than to indicate that MS was part of the causal path leading to death. Combined, these 2 categories accounted for 45%–64% of the total difference in mortality rate (compared with non-MS controls) when using the underlying and the immediate COD methods. By contrast, with the principal COD method, these uninformative classification categories accounted for only 30% of the difference in mortality. Indeed, even for other chronic disease states, these particular COD categories provide very little information about what actually caused the death and, thus, the principal COD method would be usefully applied to these other chronic diseases. In addition, this method will give practitioners better insight as to how the care of their patients might be improved in the future.

These observations are mostly consistent with previously published experience, although there are differences in the methodologies that have been used in different studies. For example, in a report from the Danish MS Registry, the authors found that in 82% of deaths MS was listed as an underlying or contributing cause and, in 56.4% it was listed as the underlying cause. [2] By contrast, the category of “infectious and respiratory diseases” accounted for only 4.7% of the deaths in their study. [2] This apparent reversal in the relative importance of MS as a cause of death when comparing the Danish cohort with the results of the present study, as discussed earlier, likely reflects the non-informative nature of MS as an underlying cause and underscores the greater insight provided by our use of the “principal” COD for categorizing deaths in MS patients. Using a different methodology from ours, a retrospective cohort study from Great Britain also reported results similar to ours. [13] That study compared mortality in MS patients with that experienced by a matched control group, and reported that MS patients had an excessive mortality due to pulmonary infections, other pulmonary disease, and cardiovascular diseases. [13] Lastly, in the 21-year long-term follow-up of MS patients from the pivotal interferon beta-1b trial, 98.4% of the original patients were identified 21 years after the start of the randomized controlled trial (RCT), and over this interval 22% (81/366) of the patients had died. [14] Of the adjudicated deaths 78.3% (54/69) were found to be MS-related. [14] Patients receiving placebo during the RCT experienced a greater all-cause mortality rate compared with those patients on active therapy, and this excessive mortality was largely due to MS-related causes, especially pulmonary infections. [15].

In our study, we used an insurance claims database to study survival patterns in MS, an approach which has potential advantages and disadvantages compared with other design strategies. Its main advantage (particularly in the US) is that, in such databases, standardized ICD10 codes are widely applied so that data is easily collated and analyzed. Moreover, in the US, death reports are obtained from 1 national source (the NDI), which has a specific format (Figure 1), and which can be easily linked to the claims database. Finally, because the population sizes are huge, the use of a claims database provided excellent statistical power.

There are, however, potential disadvantages as well. First, in the OIR data, the diagnosis of MS was based on the person having either 2 MS diagnostic ICD9 codes or 1 such code plus a prescription for a DMT. The diagnosis was not based on a review of the medical record or an examination of the patient. Nevertheless, the presence an ICD9-340 code together with a DMT prescription (which was present in 64% of patients with MS) seems to be conclusive evidence of a patient’s physician’s belief that the patient had MS. However, in the remaining 36% (where the DMT prescription is lacking) the diagnosis may be less secure. We, therefore, specifically conducted a chart review in a cohort of 85 such patients and confirmed that the diagnosis was correct in 73%. This observation suggests that the *maximum* proportion of false positives in our study population is 10% (or less) of the total. This degree of diagnostic uncertainty would not materially affect our findings.

Second, in claims data, patients were not identified at either diagnosis or onset but rather only after their enrollment in the insurance plan. Therefore, claims data do not include either the diagnosis date or the disease-onset date, so that survival can only be measured from birth rather that from those time points, which have been the principle focus of most earlier reports. [2], [4]–[6] Nevertheless, both cases and controls had to survive until plan enrollment; they were age- and sex-matched so that both cases and controls survived to the same chronological age to be included. Moreover, cases and controls were also matched on their first opportunity to die and, consequently, immortal time bias [16] cannot account for our findings.

Third, because the mean age at the first MS diagnostic code was 44 years, we commenced our follow-up of the patients with MS, on average, 15 years into their illness. [1] Consequently, our study cannot answer the question of which CODs are important for patients with MS who experience very early mortality. Nevertheless, because the vast majority of patients with MS survive substantially beyond 15 years from the onset of their disease, [2], [4]–[6] this potential gap in our data is probably of little practical consequence.

Fourth, the relatively brief period of enrollment in the health plans did not allow for a detailed measurement of comorbidities. While it would be desirable at some point to evaluate the impact of other diseases on CODs in patients with MS, the available data were insufficient for a rigorous assessment. And finally, because the patients with MS from the claims data were all commercially insured, they are representative of the commercially insured population and probably not of the total MS population in the US. Therefore, one must exercise caution when extrapolating the present results to other settings.

In summary, the principal COD method provided an improvement over other methods of using death-certificate data to determine COD, such as the underlying or the immediate COD methods. By partially resolving the uninformative category of “death due to MS” and largely resolving the uninformative category of “death due to cardiopulmonary arrest,” this method focuses attention on the excessive deaths due to other causes (Figures 3 and 4). Most conspicuously, these causes include infections (both pulmonary and genitourinary as well as sepsis) and aspiration pneumonias (Figure 4), which can be seen as expected complications in bed-bound patients. Similarly, the excessive numbers of deaths from ischemic heart disease and embolic disease may represent the consequence of immobility in advanced-stage patients and their inability to exercise. Also the greater number of deaths from accidental respiratory obstructions (Figure 4) may reflect the common impairment of swallowing, which is known to accompany the later stages of MS. [17] Thus, these particular complications seem likely to be the consequence of advanced stages of MS. Nevertheless, interestingly, excessive deaths were also observed in other disease categories (eg, accidental poisoning), which have a less obvious connection to advanced disease (Figure 4). The basis for this observation is not known. Moreover, because the principal COD method provides better insight into the actual conditions that lead to death in MS patients, it will be useful in assessing (over time) the impact of therapies (either DMTs or aggressive treatment of infections) on mortality in MS. In addition, a greater awareness of the common causes for the excessive death rate that MS patients experience (eg, fatal pulmonary infections, sepsis, and aspiration) will allow physicians to anticipate potential problems and, thereby, improve the overall care of their patients with MS.
